# DigitalMe: a journey towards personalized health and thriving

**DOI:** 10.1186/s12938-018-0553-x

**Published:** 2018-09-06

**Authors:** Sally Okun, Paul Wicks

**Affiliations:** PatientsLikeMe, 160 Second Street, Cambridge, MA 02142 USA

**Keywords:** Digital health, Omics, eHealth, mHealth, dHealth, Personalized health, Thriving

## Abstract

The use of information and communication technologies for health (eHealth) delivered via mobile-based or digitally enhanced solutions (mHealth) have rapidly evolved. When used together across various mobile applications and devices eHealth and mHealth technologies have the ability to passively monitor behavior as an indicator of socialization and mood; accumulate a range of biomedical data such as weight and heart rate; and track metrics associated with activities including steps taken and hours slept. Yet, these technologies are insufficient for measuring the full array of data about an individual and the impact of that data on a person’s current and future health. Digital health converges eHealth and mHealth with patient data about their health, healthcare, living, and environment with genomics. An innovative opportunity to unravel the complexities of disease and aging is increasingly possible with an integrative multi-omics approach informed by multidisciplinary sciences including medicine, design, biomedical informatics and engineering. The digitization of individual level data from all available sources makes possible the development of DigitalMe™, a personalized virtual avatar of a real person. The combination of longitudinally collected person generated data and molecular data derived from biospecimens offers researchers unique opportunities to better understand the mechanisms of disease while advancing person-centric hypotheses generation related to treatments, diagnostics, and prognostics.

## Background

The experience of health is unique to each individual, yet health care has traditionally focused on what is known about the disease rather than what is known about the *person with the disease*. As the use of information and communication technologies for health (eHealth) delivered via mobile-based or digitally enhanced solutions (mHealth) have rapidly evolved, the volume of personalized data reported and generated by individuals has exploded [1, 2]. While these novel data sources have the potential to monitor a person’s experiences in real time they do little to expand our knowledge about the unique characteristics of an individual that influence the response to disease, treatment or aging.

Precision medicine is an emerging frontier for biomedical discovery with the potential to significantly advance population health [3]. Precision medicine seeks to identify which treatment will be effective for which patients through long-term study of genetics, environmental and lifestyle data. In recent years significant resources have been invested in a range of public and private precision medicine initiatives [4]. The National Institute of Health’s *All of Us* Research Program [5] seeks to enroll one million Americans with an emphasis on reaching diverse and underserved populations; Project Baseline [6] is a collaboration of Google’s Verily and academic centers at Duke and Stanford Universities that will study 10,000 people of different ages, backgrounds, and medical histories over 4 years; Arivale [7], a wellness company with its roots in the One Hundred Person Wellness Project at the Institute for Systems Biology offers a concierge-style approach to genomic testing with individualized coaching; Human Longevity [8], offers high-end direct to consumer genomic testing services with plans to combine machine learning with a comprehensive database of human genotypes and phenotypes subjected to develop new ways to fight diseases associated with aging; and Foundation Medicine in partnership with Flatiron Health [9], while not focused directly on reaching patients and consumers, are developing an oncology clinic-genomic database resource for use by researchers to accelerate the development of targeted and immunotherapies to treat cancer.

A key characteristic of all precision medicine programs is the collection and biobanking of biospecimens for genomic analysis. Since the completion of the Human Genome Project in 2003 the cost of genetic testing has dropped significantly increasing access to information about specific genetic variants and genes to researchers as well as the consumer market [10]. Technological advances have resulted in efficient high-throughput analyses allowing study of the complete set of genetic material and establishing genomics as the first ‘omics discipline. Yet to truly personalize health to the individual moving beyond single ‘omics studies to a multi-omics approach is needed [11]. Personalized health will require the convergence of data from an individual’s lived experiences, family, medical and social history with the science of multiple disciplines to fully understand the environmental, biological and molecular complexity that underlies disease and aging.

## Case study: PatientsLikeMe

PatientsLikeMe (PLM) was borne out of the experience of the Heywood family when Stephen Heywood was diagnosed with amyotrophic lateral sclerosis (ALS) at the age of just 29 in 1998 [12]. There was a dearth of information available to answer their questions and much of the research related to Stephen’s diagnosis was inaccessible or impossible to replicate. Importantly, they identified a critical gap in knowledge about the lived experiences and outcomes of people with ALS because the data were not being systematically collected or shared. They believed that the right technology platform would allow patients to know all the choices they had in front of them and what happened to everyone “like me” who made those choices themselves.

Launched in 2004, the patient-facing research platform anchored within a social network seeks to improve the lives of patients through the generation of new knowledge derived from shared real world experiences and outcomes. Data reported and generated by patients using online tools systematically and longitudinally collects information about the journey of illness including pre-and post-diagnostic symptoms including timing of onset, severity and resolution; medication effectiveness, side effects and adherence. All patients are asked to report on five cross-cutting or core symptoms—fatigue, pain, insomnia, depressed mood, and anxious mood—along with other symptoms specific to their health conditions. The platform gives voice to a person’s story that is codified into data using clinically relevant terminologies and visualized meaningfully in a personalized profile that is viewable by all members of the PLM community. PLM, designed to be an open and transparent community, allows members to track their own experiences as well as share, connect and learn from others. Periodically, PLM members are invited to participate in surveys to answer specific research questions. Studies have shown that members of PLM report tangible benefits from the connectedness and sharing that is part of the PLM community experience [13, 14]. For example, in a study of military veterans living with seizures, members reported statistically significant improvements on a validated measure of self-efficacy and self-measurement after 6 weeks of site use [15].

To date, more than 650,000 PLM members have contributed 43 million data points about their real world experiences related to more than 2900 different health conditions. The more densely populated PLM communities represent life-changing conditions that may lack curative or disease modifying therapies and often require considerable self-care management. Many conditions such as fibromyalgia, multiple sclerosis, rheumatoid arthritis, systemic lupus erythematous, Parkinson’s disease, epilepsy, ALS, major depressive disorder, and post-traumatic stress disorder are chronic in nature and may progress over time. Membership in PLM is free, and patients join the network with the explicit understanding that any data they contribute will be shared anonymously for research purposes [16]. Members can also participate in the site’s online forums, where additional perspectives may be collected and analyzed through qualitative data analysis and natural language processing approaches such as probabilistic topic modeling [17]. PLM’s research mission is to conduct participatory research with its members often in collaboration with external researchers from industry [18], academic [19] and clinical centers [15] and government and non-governmental agencies including the U.S. Food and Drug Administration (FDA) and the National Quality Forum [20].

PatientsLikeMe utilizes numerous methods to collect information from patients and caregivers about their experiences including an interview process heavily influenced by ethnographic interviewing. These interviews provide rich content and have been used for the development of a patient-caregiver journey framework and patient-informed principles for measurement and design [21].

A fundamental principle articulated by patients is to be seen as more than their disease, to be seen as a whole person. This challenged PLM to develop a whole person measurement and data collection model that focuses on how patients define health—“how well my body and mind are doing” and how patients define what it means to thrive—“how well I’m living the life I want.” To meet this challenge PLM could no longer depend solely on patient reported and generated data.

## iCarbonX and Digital Life Alliance

In 2016 PLM entered into a partnership with the digital life company iCarbonX (iCX) [22]. Led by genomicist Jun Wang, iCX has created the Digital Life Alliance, an expanding ecosystem of health technology and application companies that are collaborating to digitize and analyze all aspects of life [23]. The alliance’s ultimate aim is to apply next generation biological measures and machine learning to accelerate a deeper understanding of the basis of human health and disease within the context of the patient’s lived experience.

PLM’s patient-facing research platform forms the foundation for engaging patients with the Alliance’s advanced technologies that aim to integrate and digitize many sources of patient data and biospecimens using machine learning and advanced high-throughput scientific technology for multi-omics analyses of RNA, DNA, proteins, antibodies, microbiome and metabolites. The Digital Life Alliance aims to leverage the digitization of person generated experiential data, biotechnology, artificial intelligence and machine learning with the long-term vision of creating a mobile based digital avatar that is able to guide the user toward a range of healthy life options and interventions that are personalized to the outcomes that matter to the individual.

The value of PLM as a research-based social network for patients and caregivers is realized through aggregated knowledge derived from shared real world experiences of others [24]. These experiences, when expressed in de-identified data form the foundation of the PLM business model of creating value for diverse stakeholders interested in accessing insights from the patient perspective. The addition of biospecimen collection and analysis to the PLM platform will eventually offer patients direct and indirect benefits in the form of individual and population-based findings. As the PLM data model matures commercial applications will be developed to support the company’s evolving business model. These consumer applications will be guided by the company’s policies for privacy [16], transparency and openness [25], all hallmarks of the PLM brand.

## Measurement model of health and thriving

DigitalMe Ignite, a PLM pilot study launched in June 2017, will integrate methods to measure how well an individual’s body and mind are doing (health) and how well an individual is living the life she wants (thrive). The protocol approved by the New England Institutional Review Board (NEIRB) is enrolling controls and several hundred patients with conditions in three therapeutic areas: neurology (ALS, multiple sclerosis, Parkinson’s disease), mental health (major depressive disorder, bipolar, post-traumatic stress disorder) and autoimmune/rheumatology (systemic lupus erythematous, rheumatoid arthritis, fibromyalgia). The participant experience includes blood specimen collection at the time of enrollment and every 4 months thereafter to interrogate the biological state with deep molecular profiling.

The health conditions were specifically selected for their syndromic characteristics that often involve periodic or episodic exacerbation, relapse, flare or other changes from a baseline state identified through measurement and/or by the patient herself. State changes prompt the collection of a blood sample and additional patient experience data. Research question clusters associated with state changes will be studied across different conditions and different complexity levels to better understand the patient experience and the biological profile during the episode. Specific research questions will evolve but may include questions such as, “Are there biological outliers who do not look similar to others with the same condition? What is similar and what is different? Why?”; “Can understanding the molecular signature of an individual’s disease over time help define the clinical parameters of a disease more clearly?”; and “Is there a relationship between the molecular characteristics of an individual’s disease and the individual’s expression of her function and feeling?” While these questions are necessarily broad, more specific hypothesis will be possible to test as data comes in.

PLM researchers developed a new patient-reported measure that could be used across conditions for all members of PLM. The measure, known as Thrive™, integrates the conceptual framework of the International Classification of Function and Disability and Health [26] (ICF) as a starting reference point since the model reflects the diversity of domains relevant to living with illness including body function and structures, activity and participation, environmental factors, and personal factors. The Thrive measure includes three sections that will be mapped to the ICF framework for reference and include: (1) health and symptoms, (2) how well you can do what matters (functioning), and (3) how you’re feeling about it (thriving). The result is a scalable, computable approach that incorporates common measurement domains plus cohort specific measurement domains based on conditions, treatments or other attributes of the condition.

Validation of Thrive has completed the qualitative phase of concept elicitation drafted from existing scales, the ICF framework, and feedback interviews [27]. The first quantitative and psychometric phase included testing the Thrive version 1.0 with 2002 patients, followed by a 3-day retest with over 924 patients, followed by a 30-day retest with 717 patients [28]. A second quantitative and psychometric phase of Thrive version 1.1 was completed using the same test–retest timeframes with 704, 239 and 49 patients respectively.

Participants enrolled in DigitalMe Ignite since its launch are completing the Thrive measure at specific points in time associated with blood specimen collection. Also, in September 2017 it was deployed to all patients across the PLM site with expectations that it be completed every 30 days. Additionally, Thrive plus modules specific to systemic lupus erythematous and blood collection will be used in a 30-month clinical validation study at an academic medical center starting in early 2018.

The integration and digitization of all data related to an individual represents a significant shift from PLM’s roots as a platform that collected data solely provided by patients themselves. PLM created the DigitalMe Ignite protocol by building upon well-established research and scientific operating procedures already in place including data protections and a designated for human protection administrator. PLM works closely with NEIRB, an external institutional review board, to ensure appropriate oversight of all aspects of the DigitalMe Ignite protocol. PLM is convening an Ethics and Compliance Advisory Board (ECAB) that includes thought leaders, subject matter experts and patients. The inaugural meeting of the ECAB charter members was held in March 2018 at which time three members were confirmed including the ECAB Chair. Nominations to fill six to seven additional seats were solicited in June 2018 and formal letters of invitation were sent in August 2018 after an internal review process that included the ECAB Chair. The full ECAB will convene for an in-person meeting in October 2018.

PLM’s long-standing core values of putting patients first, openness, transparency and honoring members trust are perfectly aligned with the inspiring principles of DigitalMe—to create a personalized learning health system that helps individuals make better informed decisions about their current and future health based on the aggregate learning of the whole community.

## Imagine: the future of health

Imagine the future of health in one’s own hands. Imagine a future where a patient can provide her clinician relevant differential diagnostic data based on algorithmically driven evidence from her digital doppelgänger. Imagine a future where one’s DigitalMe avatar is the ‘guinea pig’ upon which a new treatment is tested for effectiveness before the patient and her clinician decide on a course of treatment. This vision of the future of health is ambitious and the journey towards it is just beginning for PLM and the many precision medicine initiatives now underway [5–9].

DigitalMe requires collaborative and respectful alignment across diverse sciences, ecosystems and people. The rapid pace of technological advances in biomedicine, computational biology, biomedical engineering, and machine learning will no doubt test the boundaries of cultural, ethical, and societal norms. The journey ahead requires an incremental collaborative approach akin to crossing the river by feeling the stones to successfully reach the intended destination—a future that helps each of us achieve personalized health to know how well our mind and body are doing—and personalized thriving to gauge how well we are living the life we want.
